# Ozone-Mediated Modulation of Green Tea Extract Enhances Bioactive Compounds and Therapeutic Potential Relevant to Human Health

**DOI:** 10.3390/ph18111694

**Published:** 2025-11-08

**Authors:** Abdulrahman S. Bazaid, Sulaiman A. Alsalamah, Husam Qanash, Mohammed Ibrahim Alghonaim, Nizar H. Saeedi, Abdu Aldarhami

**Affiliations:** 1 Department of Medical Laboratory Science, College of Applied Medical Sciences, University of Ha’il, Hail 55476, Saudi Arabia; 2 Department of Biology, College of Science, Imam Mohammad Ibn Saud Islamic University (IMSIU), Riyadh 11623, Saudi Arabia; saalsalamah@imamu.edu.sa (S.A.A.); mialghonaim@imamu.edu.sa (M.I.A.); 3 Medical and Diagnostic Research Center, University of Ha’il, Hail 55473, Saudi Arabia; 4 Department of Medical Laboratory Technology, Faculty of Applied Medical Sciences, University of Tabuk, Tabuk 71491, Saudi Arabia; nsaeedi@ut.edu.sa; 5 Department of Microbiology and Parasitology, Faculty of Medicine, Umm Al-Qura University, Al-Qunfudah 21961, Saudi Arabia; ahdarhami@uqu.edu.sa

**Keywords:** ozone, green tea, phenolic compounds, pathogenic microbes, biological properties

## Abstract

**Background**: Ozonation is a non-thermal process that can remodel the chemistry and bioactivity of plant extracts. We evaluated whether ozonating green tea extract enhances its phenolic composition and in vitro bioactivity in relation to nutrition and food applications, with potential clinical applications. **Methods:** Ethanolic green tea extract (GTE) was exposed to ozone (0–7 L/min, 5 h) to yield an ozonated extract (GTOE). Phenolics were quantified by the HPLC. Bioactivities included antimicrobial testing (agar diffusion; MIC/MBC/MFC), antibiofilm formation, time-kill kinetics (0–180 min), bacteria-induced hemolysis in human RBCs, DPPH radical scavenging, pancreatic lipase inhibition, and scratch-wound closure in human fibroblasts. Data from *n* = 3 independent experiments were analyzed by one-way ANOVA with Tukey’s post hoc test (α = 0.05). **Results**: Ozonation increased gallic acid (3150.92 to 3229.69 µg/g) and ellagic acid (2470.66 to 2789.40 µg/g), while catechin decreased slightly (2634.09 to 2535.09 µg/g). Compared with GTE, GTOE produced larger inhibition zones and lower MIC/MBC/MFC against *Candida albicans*, *Bacillus subtilis*, *Staphylococcus aureus*, *Klebsiella pneumoniae*, and *Salmonella typhi*; *Aspergillus niger* remained unsusceptible. For example, inhibition zones for *S. aureus* and *K. pneumoniae* increased by 2–4 mm and MIC/MBC values were 2-8-fold lower. *Candida albicans* showed marked sensitivity (MFC 500 to 125 µg/mL). GTOE exhibited superior, dose-dependent antibiofilm activity across all tested strains, reaching up to 97.82% inhibition, (highest for *S. aureus* and *S. typhi*, at 75% MBC). GTOE reduced bacterial counts more rapidly than GTE across all tested strains, achieving full eradication within 150 min. Bacteria-induced hemolysis was inhibited by 97% at 75% MIC with GTOE, versus 93–96% with GTE. Antioxidant capacity improved (DPPH IC_50_ 3.31 vs. 5.54 μg/mL), as did lipase inhibition IC_50_ 6.06 vs. 17.69 μg/mL). Wound closure at 48 h increased (GTOE 61.1%; GTE 56.8%; control 50.8%). **Conclusions**: Controlled ozonation of green tea extract remodeled phenolics and consistently enhanced antimicrobial, antibiofilm, antioxidant, potential anti-obesity, and wound-healing activities in vitro. These results support food-grade optimization and safety/by-product profiling, followed by in vivo validation at diet-relevant doses, to enable nutrition, food, and potential clinical applications.

## 1. Introduction

Green tea (*Camellia sinensis*) is among the most widely consumed beverages worldwide. It has long been recognized for its health-promoting properties due to its rich content of bioactive compounds. As antibiotic-resistant pathogens continue to rise worldwide, natural products, including green tea, are gaining attention as promising sources of therapeutic antimicrobial agents [1]. The main constituents of green tea, including polyphenols, caffeine, amino acids and antioxidants, determine its physicochemical characteristics. These components are associated with its pH level, antioxidant capacity, and antibacterial activities [1]. Polyphenolic molecules are the main constituent of green tea, which are a class of antioxidants known as catechins, including epicatechin gallate (ECG), epigallocatechin gallate (EGCG), and epicatechin (EC). The specific configurations of galloyl and hydroxyl groups in these compounds control their biological activity, involving their antioxidant and hydrogen-bonding properties. According to Luo et al. [2], the composition of green tea also includes other polyphenols such as phenolic acids and flavanols (such as quercetin) [2]. It contains a diverse range of biologically active substances, including polyphenols (especially catechins), amino acids, polysaccharides, alkaloids, terpenoids, macro minerals, trace elements, and vitamins [2]. These compounds are linked to multiple therapeutic effects, such as antioxidants, anticancer, anti-inflammatory, anti-obesity, and anti-diabetic activities, as well as cardiovascular benefits and modulation of immune responses and gut microbiota [2]. Previous studies have reported the positive effects of ozone exposure on the phytochemical composition and quality of cultivated green tea [3,4]. Generally, plant-derived natural products have shown promising antimicrobial efficacy against resistant microbes. Green tea in particular is a rich source of compounds with therapeutic potentials [5,6,7,8]. In addition, green tea has shown promise in managing obesity by increasing metabolism and promoting fat burning [9]. Obesity is a major risk factor for several chronic diseases, including metabolic syndrome, cardiovascular disorders, and type 2 diabetes [10]. Oxidative stress is a pathological condition that results from an imbalance between the generation of reactive oxygen species (ROS) and the body’s antioxidant defense systems [10]. Excessive ROS production can damage cellular structures, including lipids, proteins, and DNA, contributing to the development of numerous diseases such as cancer, diabetes, neurodegenerative disorders, and cardiovascular diseases. In this context, green tea has gained considerable attention not only as a dietary supplement but also as a functional ingredient with broad bioactivities, including antimicrobial, antioxidant, anti-obesity, and wound healing effects [11,12].

Although green tea’s phytochemical richness is well established, its composition can vary significantly depending on factors such as geographical origin, harvest time, processing, and storage conditions [13,14]. These factors affect both the qualitative and quantitative profiles of biologically active substances (BAS) and influence the sensory properties of the final tea product [15]. Recently, biotechnological approaches have been explored to enhance the pharmacological potential of plant extracts and oils. Techniques such as UV exposure [16] and ozonation [17] have been studied for their ability to improve biological activities. Ozone (O_3_), a triatomic oxygen molecule and strong oxidizing agent, has been applied in multiple fields because of its antimicrobial and oxidative properties [18]. Ozonated oils, for example, exhibit modified chemical compositions and significantly enhanced antioxidant and antimicrobial activities [19].

Despite the promising results of ozonation on oils [19,20], limited scientific evidence exists regarding how ozone treatment influences the phytochemical composition and therapeutic efficacy of plant-based aqueous extracts such as green tea. Although previous studies have examined the general effects of ozonation on various plant materials, there is still limited evidence regarding its specific influence on the phytochemical composition and functional properties of plant extracts and their oils. To address this gap, the present study explores ozone-mediated modulation in green tea extract and establishes a direct link between ozone-induced chemical changes and enhanced multifunctional therapeutic potential, including antimicrobial, antioxidant, potential anti-obesity (inferred from pancreatic lipase inhibition), and wound-healing activities. This study provides novel insights into ozonation as a safe, non-thermal approach to improve the functional quality of green tea for pharmaceutical, nutraceutical, and cosmeceutical applications by correlating ozone-induced phytochemical changes with alterations in bioactivity.

## 2. Results and Discussion

### 2.1. Phenolic and Flavonoid Profile of Green Tea Extract

**Figure 1:**
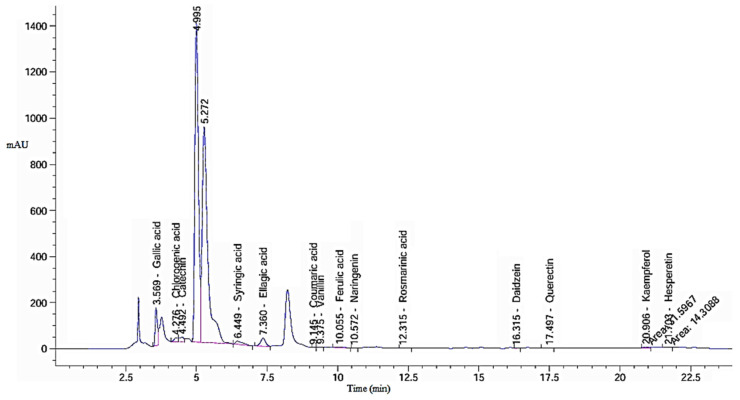
Chromatogram of HPLC analysis of Ethanolic green tea extract (GTE).

**Figure 2:**
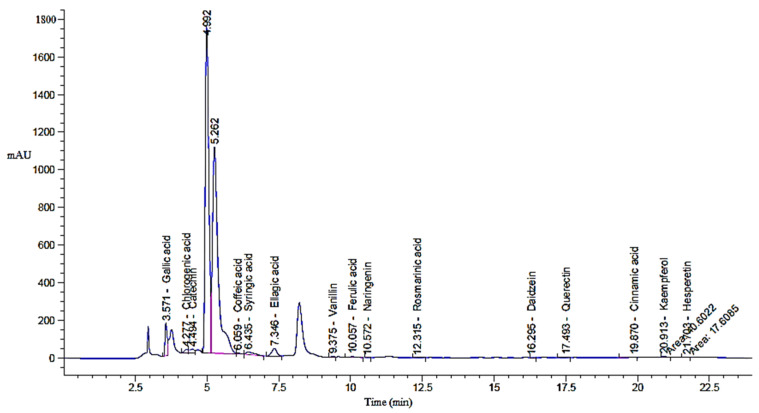
Chromatogram of HPLC analysis of GTOE.

A total of 6.0 gm of green tea powder was ozonized, yielding equivalent amount of ozonized extract, which was tested in various experiments. The result shows the HPLC profiles of key phenolic and flavonoid compounds identified in both green tea extract (GTE; Figure 1) and its ozonized counterpart (GTOE; Figure 2), compared with polyphenolic standard (Table 1).

Ozonation substantially affected both the qualitative and quantitative profiles of the bioactive compounds. Among the predominant constituents gallic acid, catechin, and ellagic acid were detected at relatively high concentrations in both extracts. Gallic acid slightly increased in GTOE (3229.69 µg/g) compared to GTE (3150.92 µg/g), suggesting a mild oxidative release or preservation effect during ozonation. A similar trend was observed for ellagic acid, which rose from 2470.66 µg/g in GTE to 2789.40 µg/g in GTOE, indicating that ozone may have enhanced liberation from bound forms or increased extractability.

Significant increases (*p* ≤ 0.05) were also noted for gallic acid, caffeic acid, coumaric acid, vanillin, and cinnamic acid in GTOE compared with GTE. Conversely, catechin, one of the most abundant and biologically active flavonoids in green tea, showed a slight reduction in GTOE (2535.09 µg/g) versus GTE (2634.09 µg/g), reflecting its potential susceptibility to ozone-induced degradation. A notable reduction (*p* ≤ 0.05) in coumaric acid was also observed GTOE relative to GTO, consistent with the fact that catechins account for approximately 70% of tea polyphenols and are the principle active constituents of tea [21].

Compounds such as rosmarinic acid, quercetin, kaempferol, and hesperetin were detected in both extracts, with a relative increase in GTOE, indicating ozone stability of these flavonoid structures. For instance, quercetin remained fairly stable (112.65 µg/g in GTE vs. 122.94 µg/g in GTOE), and hesperetin increased modestly after ozonation (33.83 µg/g vs. 41.63 µg/g). These findings are significant, as stable phenolics retain their bioactivity even after oxidative treatment.

Notably, caffeic acid and cinnamic acid were absent in GTE but appeared in GTOE. This emergence may be attributed to ozone-mediated breakdown of larger polyphenols or glycosidic conjugates, resulting in smaller, structurally simpler antioxidant compounds. A report showed that green tea contains various compounds such as gallic acid, chlorogenic acid, p-coumaric acid, caffeic acid, ellagic acid and quinic acid [22]. Ozonation typically triggers oxidative reactions that generate new oxygen-rich molecules with improved biological activity [23]. It is also noted that ozonation can modify certain bioactive molecules while preserving others, potentially retaining their therapeutic properties [24].

Nevertheless, given that dietary phenolic and flavonoid compounds are widely recognized as safe for human consumption [25,26], the present study focused on evaluating their ozone-induced functional enhancement rather than toxicity. Future research will address cytotoxicity, pharmacokinetics, and product stability to support potential clinical and/or nutraceutical applications.

### 2.2. Antimicrobial Efficacy of Green Tea Extract

Table 2 summarizes the antimicrobial activity of GTE and GTOE against various bacterial and fungal pathogens, assessed by the zone of inhibition (mm), MIC, and MBC or MFC values. Across all tested organisms, GTOE consistently produced larger inhibition zones and lower MIC and MBC/MFC values than GTE, indicating improved antimicrobial potency after ozonation.

For the bacterial strains, *B. subtilis* showed an increase in the inhibition zone from 13 ± 0.1 mm (GTE) to 16 ± 0.3 mm (GTOE), while MIC decreased from 31.25 ± 0.4 μg/mL to 15.62 ± 0.2 μg/mL and MBC from 125 ± 0.6 μg/mL to 31.25 ± 0.1 μg/mL. *Staphylococcus aureus* exhibited a similar pattern, with the inhibition zone increasing from 19 ± 0.4 mm to 21 ± 0.7 mm and both MIC and MBC values decreasing fourfold. In *Klebsiella pneumoniae*, the zone of inhibition increased from 16 ± 0.6 mm to 20 ± 0.5 mm, MIC dropped from 62.5 ± 0.3 to 15.62 ± 0.3 μg/mL and MBC decreased sharply from 125 ± 0.6 to 15.62 ± 0.4 μg/mL. For *Salmonella typhi*, the inhibition zone expanded from 15 ± 0.8 mm (GTE) to 22 ± 0.2 mm (GTOE), while MIC and MBC both reduced by half.

Among fungal strains, *Candida albicans* showed a notable increase in the inhibition zone from 13 ± 0.3 mm to 18 ± 0.1 mm, with MIC decreasing from 62.5 ± 0.6 to 31.25 ± 0.2 μg/mL and MFC from 500 ± 0.6 to 125 ± 0.6 μg/mL after ozonation. In contrast, *Aspergillus niger* showed no inhibition with either extract.

These findings confirm that ozonation enhances the antimicrobial efficacy of green tea extract, likely by modifying or activating polyphenolic compounds to exert stronger effects against both bacteria and fungi. Saponin fractions isolated from green tea demonstrated strong antimicrobial activity against *E. coli*, *Streptococcus mutans* and multiple strains of *Salmonella* [27]. Similarly, ozonation of peanut oil significantly enhance its antimicrobial efficacy, with notable reduction (*p*  ≤  0.05) in MIC and MBC values [19]. They observed a decrease in MIC and MBC values for *Bacillus subtilis* from 31.25 ± 0.4 to 15.62 ± 0.2 µg/mL and from 62.5 ± 0.1 to 31.25 ± 0.3 µg/mL, respectively. Additionally, a comparable trend was also noted for *S. aureus*, where MIC decreased from 62.5 ± 0.3 to 15.62 ± 0.1 µg/mL, and MBC dropped from 125 ± 0.1 to 31.25 ± 0.1 µg/mL.

HPLC analysis revealed that green tea extract contained high level of catechin, which may contribute to its broad-spectrum antibacterial potential. Catechins have demonstrated activity against *S. aureus*, *E. coli*, *Pseudomonas aeruginosa*, and *Helicobacter pylori*, even in multidrug-resistant strains. It was reported that catechins can enhance the activity of conventional antibiotics, suggesting a synergistic mechanism [28].

The antimicrobial action of catechins involves multiple mechanisms, including disruption of bacterial membranes, increased membrane permeability, oxidative stress induction through hydrogen peroxide generation, and inhibition of key bacterial enzymes and gene expression. These effects collectively impair bacterial adhesion, colonization and survival [29]. Matcha green tea strongly inhibited pneumococci [30]. Polyphenol hydroxyl groups can bind to bacterial cell membrane proteins, alter permeability and disrupt membrane integrity. Moreover, tea polyphenols can regulate bacterial metabolism, inhibit intracellular enzymes activity and nucleic acids synthesis, and damage cell walls and membranes, thereby exhibiting broad-spectrum antibacterial activity [31].

### 2.3. Anti-Biofilm Activity of Green Tea Extract

**Figure 3:**
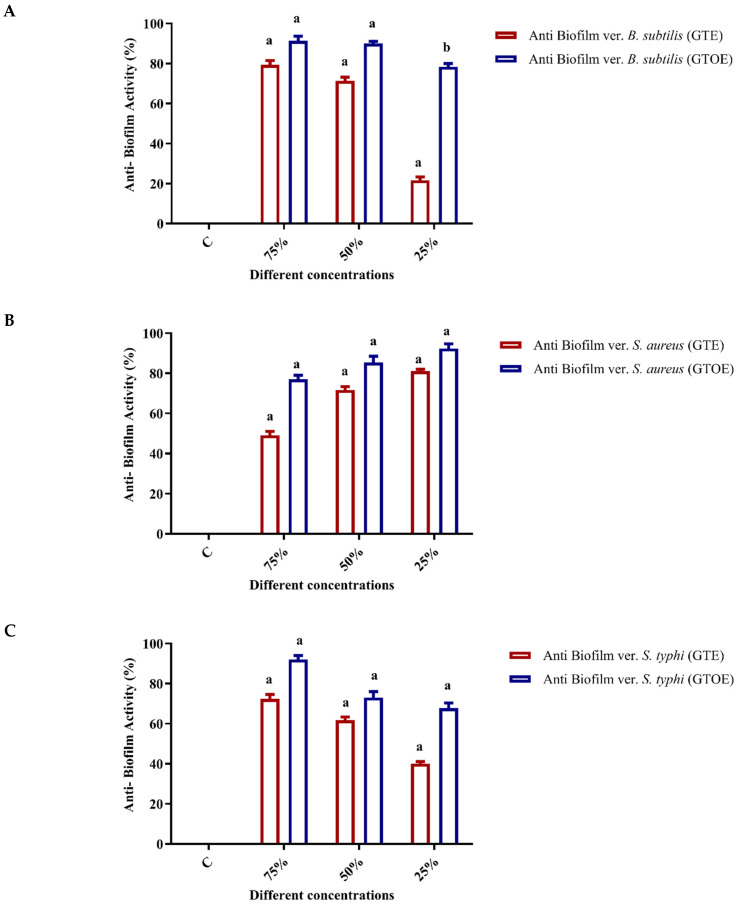

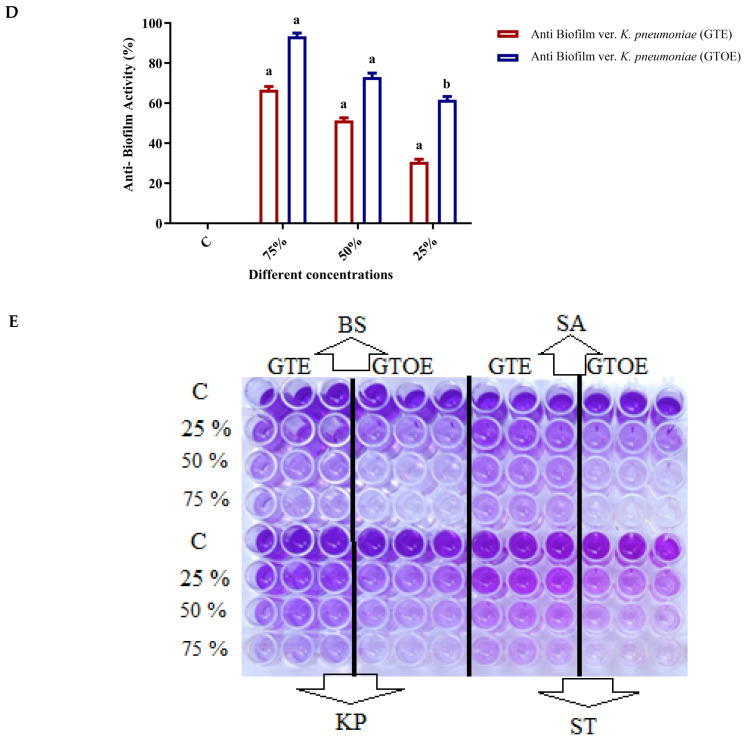
Statistical analysis of the antibiofilm activity of green tea extract (GTE) and ozonated green tea extract (GTOE) against various bacteria strains. Data are expressed as means ± SD; different letters above the columns indicate significant differences (*p* ≤ 0.05). (**A**) *Bacillus subtilis* (BS), (**B**) *Staphylococcus aureus* (SA), (**C**) *Salmonella Typhi* (ST), (**D**) *Klebsiella pneumoniae* (KP), and (**E**) crystal violet biofilm assay in a 96-well plate. *B. subtilis* (BS), *S. aureus* (SA), *K. pneumoniae* (KP), and *S. typhi* (ST).

The results from the crystal violet biofilm assay in a 96-well plate (Figure 3) demonstrated the biofilm inhibition efficacy of GTE and GTOE against *B. subtilis*, *S. typhi*, *S. aureus*, and *Klebsiella pneumoniae*, at different concentrations (25%, 50%, and 75% of the MBC). At 25% MBC, both extracts showed initial biofilm inhibition, but GTOE exhibited significantly higher activity across all tested strains (e.g., 92.05% vs. 20.49% for *B. subtilis*). At 50% MBC, inhibition markedly increased for both extracts, with GTOE maintaining consistently high activity (e.g., 91.56% for *S. typhi*, 79.67% for *S. aureus*). At 75% MBC, biofilm inhibition peaked, with GTOE outperforming GTE. The highest inhibition rates were recorded for *S. typhi* (96.12%) and *S. aureus* (97.82%) under GTOE treatment. Overall, both extracts demonstrated dose-dependent biofilm inhibition, with GTOE showing superior antibiofilm activity. This enhanced effect may result from the increased oxidative capacity introduced by ozonation, which more effectively disrupts bacterial cell walls and the biofilm matrix. Gram-positive bacteria appeared more sensitive than Gram-negative *K. pneumoniae*, likely due to structural differences in their cell envelopes.

These findings highlight the potential of ozonized green tea extract as a natural antibiofilm agent, particularly against drug resistant Gram-positive pathogens. The improvement in antibiofilm activity following ozonation may be attributed to several factors: oxidative modification of key active compounds, leading to more potent or bioavailable antimicrobial molecules [32], and the formation of reactive ozonides that interfere with bacterial enzymatic systems [33].

### 2.4. Bactericidal Kinetics of Green Tea Extract

The time-kill kinetics of GTE and GTOE against selected bacterial strains demonstrated a clear time-dependent bactericidal effect, with GTOE consistently showing enhanced activity compared with GTE (Table 3). At the initial time-point (0 min), all bacterial strains, including *B. subtilis*, *S. aureus*, *K. pneumoniae*, and *S. typhi*, exhibit high viable cell counts, ranging from 19 × 10^5^ to 38 × 10^5^ CFU/mL, with no difference between GTE and GTOE treatments. As time progresses, both extracts begin to reduce the bacterial population. In the case of *B. subtilis*, GTE reduces the count steadily, reaching 12 CFU/mL by 150 min and complete killing (0 CFU/mL) by 180 min. GTOE, however, demonstrates a more rapid effect, bringing the count down to 14 CFU/mL by 120 min and eliminating all viable cells by 150 min. A similar pattern is observed with *S. aureus*, where GTE causes a gradual decline with some viable cells (160 CFU/mL) still present at 150 min., while GTOE achieves complete killing at the same time point. For *K. pneumoniae*, GTE also reduces the bacterial load progressively, reaching 175 CFU/mL at 180 min. In contrast, GTOE shows a more potent response, decreasing the count to 36 CFU/mL at 150 min and achieving full eradication by 180 min. The most pronounced difference is seen with *S. typhi*, where GTOE leads to a sharp reduction from 25 × 10^5^ to 0 CFU/mL within 150 min, while GTE maintains a significant number of viable cells (233 CFU/mL) at that point and only reaches complete killing at 180 min. Overall, the data confirm that both GTE and GTOE are effective in reducing bacterial viability over time, with GTOE consistently providing faster and more efficient bacterial killing across all tested strains. This enhanced antimicrobial activity of GTOE may be attributed to ozonation, which likely enhances the bioavailability or reactivity of key phytochemicals within the extract, thereby improving their bactericidal potential. Ozonation of pumpkin seed oil enhanced its antimicrobial activity, where the inhibition zone and antibiofilm increased as a result of exposure to ozone against *H. pylori* [16]. Moreover, killing time assay, MIC and MBC confirmed the improved antimicrobial potential of pumpkin seed oil exposed to zone compared to non-ozonized oil.

### 2.5. Anti-Hemolytic Activity of Ozonated Green Tea Extract

**Figure 4:**
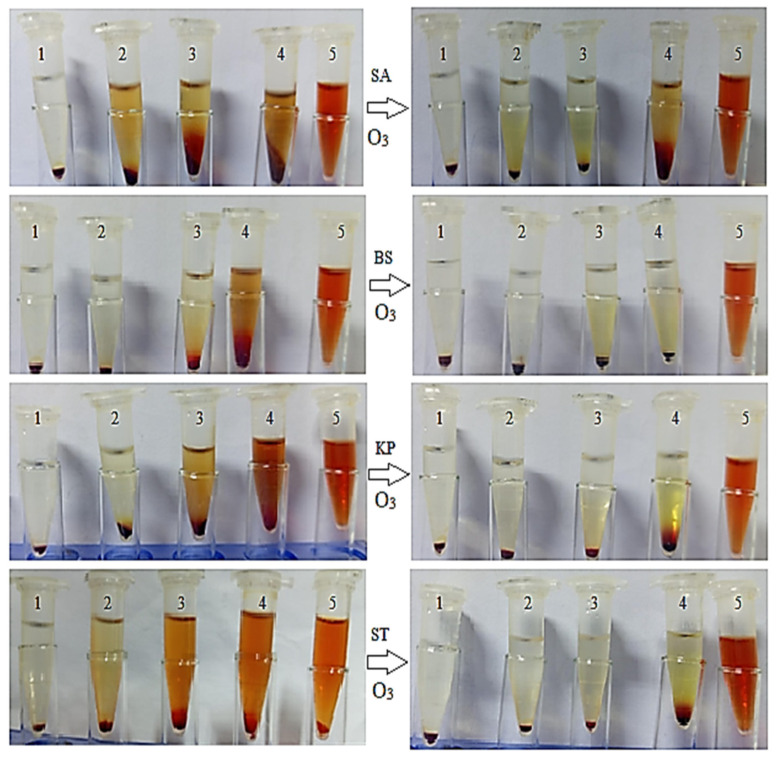
Hemolysis (%) inhibition in presence of various bacteria *S. aureus* (SA), *B. subtilis* (BS), *K. pneumoniae* (KP), and *S. typhi* (ST) treated with untreated green tea extract and exposed to ozone (O_3_). Treatments include standard drug (1), 25% MIC (4), 50% MIC (3), 75% MIC (2), untreated (5), and control complete hemolysis By SDS (5).

**Figure 5:**
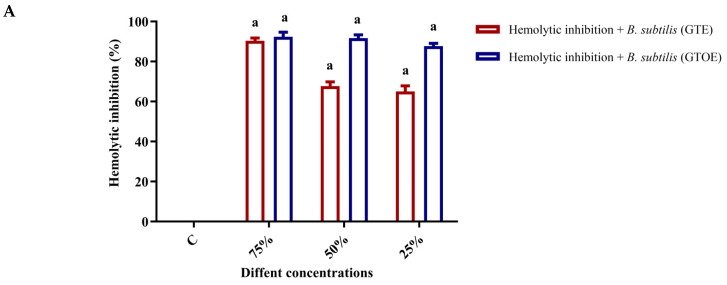

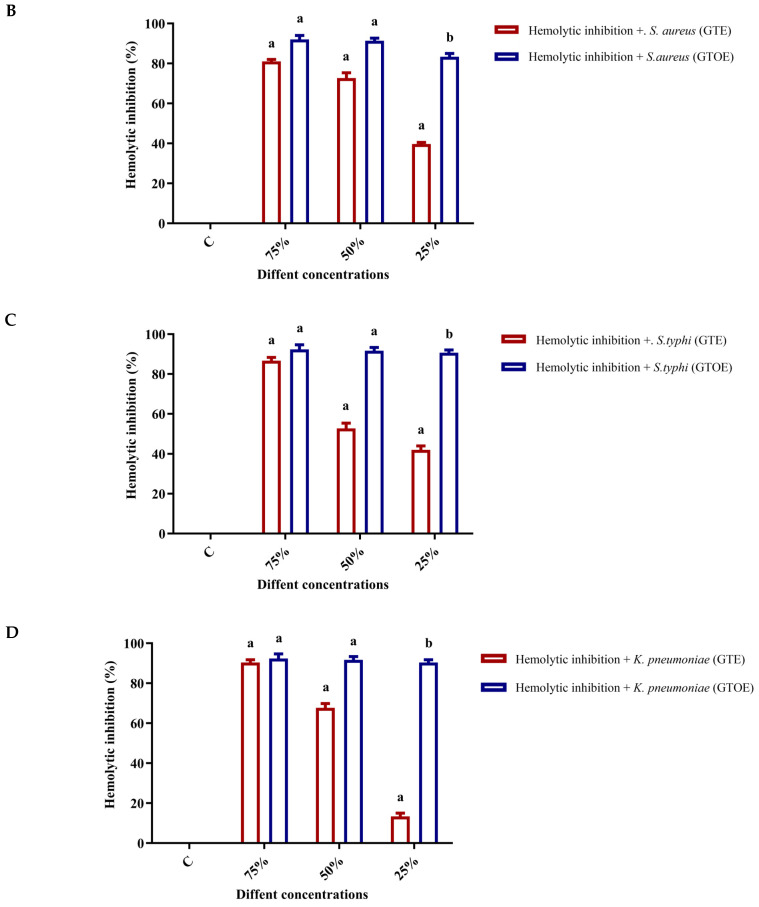
Statistical testing for hemolytic inhibition percentages of GTE and GTOE at different levels of MIC in presence of various bacteria (Data are presented as means ± SD; Various letters above columns refer to significant difference where *p* ≤ 0.05); (**A**) in presence of BS, (**B**) in presence of SA, (**C**) in presence of ST, (**D**) in presence of KP.

The image and data provided in (Figure 4 and Figure 5) illustrate the percentage inhibition of hemolysis in the presence of various bacterial strains—*B. subtilis*, *S. typhi*, *S. aureus*, and *K. pneumoniae*, when treated with GTE and GTOE at increasing concentrations (25%, 50%, and 75% of the MIC). A control showing complete hemolysis induced by SDS is included for comparison. The visual data clearly show a progressive reduction in hemolysis as the concentration of both GTE and GTOE increases, confirming a dose-dependent protective effect against erythrocyte lysis. Notably, GTOE consistently outperformed GTE in all bacterial contexts, suggesting that ozonation enhances the hemolytic inhibition potential of green tea extract. For *B. subtilis*, GTOE achieved nearly complete hemolysis inhibition (97.26 ± 1.78%) at 75% MIC compared to 95.82 ± 1.02% by GTE. In the case of *S. typhi*, the inhibition was also high for GTOE (96.9 ± 0.98%) compared to GTE (83.20 ± 0.50%) at the same concentration. Against *S. aureus*, the difference was more pronounced: while GTE only inhibited hemolysis by 46.28 ± 0.66% at 25% MIC, GTOE achieved 91.67 ± 0.04%, and this trend continued at higher concentrations. Similarly, for *K. pneumoniae*, GTOE reached 97.50 ± 1.33% inhibition at 75% MIC, while GTE reached 93.4 ± 0.66%. In summary, these findings demonstrate that both GTE and GTOE exhibit noteworthy anti-hemolytic activity in the presence of various bacteria, with the ozonized extract showing superior efficacy. This suggests potential for GTOE as a protective agent against bacterial-induced hemolysis, possibly due to enhanced antioxidant or membrane-stabilizing properties post-ozonation. In the present investigation, green tea extract consists of several phenolic and flavonoids compounds, particularly catechin. The flavonoids and catechin minimize the hemolysis via oxidative stress [34]. Also, the antihemolytic activity of green tea extract was reported because of its phytochemicals [35]. The improvement in antihemolytic activity of GTOE was in accordance was previously published work and ozonized pumpkin seed oil has better antihemolytic impact than crude oil [17].

### 2.6. Wound Healing Properties of GTE and GTOE

**Figure 6:**
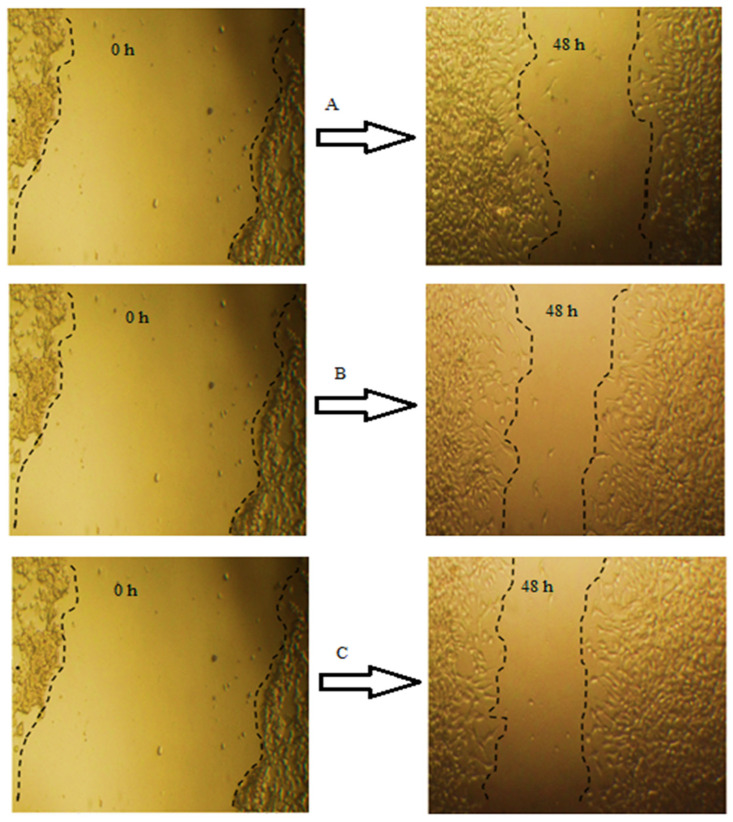
Wound Healing prosperities of GTE and GTOE using HFB4 cells; left panel: microscopic examination of wound healing using various treatments—right panel: statistical analysis for wound healing percentages upon using GTE and GTOE relative to control.

The wound healing capacity of GTE and GTOE was evaluated using a scratch assay in HFB4 human fibroblast cells (Table 4 and Figure 6). Quantitative parameters including wound width, area difference, and percentage of wound closure over a 48-h period are presented in the accompanying table and micrographs. At baseline (0 h), all groups showed nearly identical wound widths (~1462.88 µm) and areas (~1.58 × 10^6^ µm^2^), ensuring equal starting conditions. After 48 h, both GTE and GTOE treatments resulted in greater wound closure compared to the control. The control group showed 50.82% wound closure, while GTE-treated cells reached 56.77%, and GTOE-treated cells demonstrated the highest closure at 61.13%. These findings were consistent with the calculated area differences and residual wound margins (RM). GTOE-treated cells exhibited the greatest area reduction (965,500.8 µm^2^) and the widest remaining margin (18.63 µm), suggesting enhanced cellular migration and proliferation. These observations were visually confirmed by microscopic imaging at 0 and 48 h. The wound gap in the control group (image A) remained partially open, whereas GTE-treated cells (image B) displayed moderate closure. In contrast, the GTOE-treated group (image C) showed nearly complete re-epithelialization, with minimal remaining gap, consistent with the quantitative data. The improved wound healing activity of GTOE can be attributed to the ozone-induced enhancement of the phytochemical profile. As previously discussed, ozonation led to increases in several bioactive phenolic acids and flavonoids, including syringic acid, ellagic acid, gallic acid, ferulic acid, and kaempferol, which are known to stimulate fibroblast migration, collagen deposition, and antioxidant defense mechanisms. These results support the notion that GTOE not only preserves but enhances the regenerative potential of green tea extract. Its superior performance in wound closure assays highlights its promise as a natural, bioactive agent for skin healing, whether in cosmeceutical formulations or therapeutic wound care products. Further studies may explore the mechanistic pathways, such as modulation of growth factors or inflammatory mediators, underlying this enhanced activity. Tea catechins regulated the expression of antioxidant enzymes and reduced oxidative stress, thus protecting primary goat hepatocytes in vitro [36]. Due to their excellent antioxidant and antibacterial properties, tea polyphenols show great potential in wound healing. Lan et al. successfully fabricated a novel fiber membrane with a core layer of tea polyphenols and a shell layer of ε-poly (ε-PL), which are excellent wound dressings with dual delivery of antioxidant and antimicrobial delivery [37]. Furthermore, researchers reported that ozonized plant extracts can improve fibroblast migration, boost growth factors, and organize the inflammatory response, thereby enhance wound closure, and tissue repair [38,39].

### 2.7. Potential Anti-Obesity Activities of Green Tea Extract

**Figure 7:**
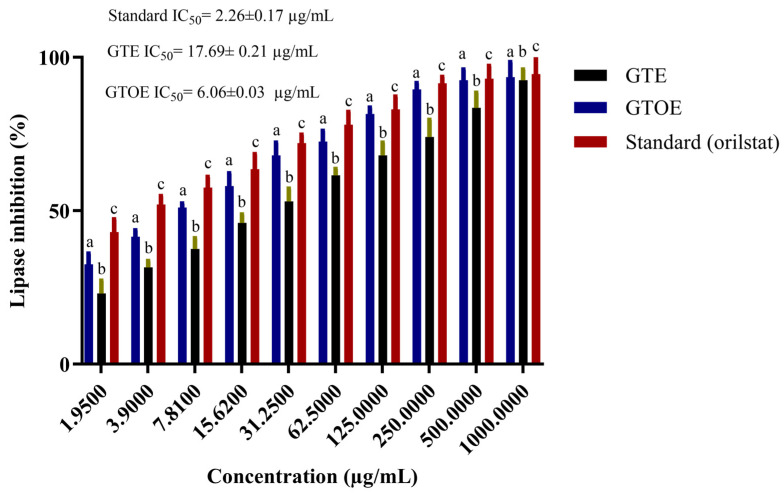
Statistical testing for lipase inhibition percentages for GTE, GTOE versus standard (Data are represented as means ± SD; different letters above the columns indicate statistically significant differences (a vs. b: *p* ≤ 0.05; b vs. c: *p* ≤ 0.05; a vs. c: *p* ≤ 0.01).

Figure 7 illustrates the concentration-dependent effects of GTE and GTOE on pancreatic lipase inhibition and DPPH radical scavenging, benchmarked against Orlistat and ascorbic acid, respectively. At all tested concentrations, GTOE consistently demonstrated superior lipase inhibitory capacity over unmodified GTE. This effect became particularly pronounced beyond 15.62 µg/mL, where GTOE surpassed 60% inhibition and continued rising to over 95% at 1000 µg/mL. In contrast, GTE required higher doses to achieve comparable inhibition, indicating reduced potency. Notably, Orlistat maintained the highest activity at most concentrations, but GTOE closely approached its efficacy at elevated doses. The trend in IC_50_ values reinforces this observation. GTOE achieved a markedly lower IC_50_ (6.06 µg/mL) than GTE (17.69 µg/mL), reflecting increased potency, potentially due to chemical alterations of bioactive constituents through ozonation. Although Orlistat remained the most potent inhibitor (IC_50_ = 2.26 µg/mL), the performance of GTOE highlights its therapeutic promise as a natural alternative for managing dietary fat absorption. In this case, the GTOE exhibited markedly stronger lipase inhibitory activity compared to GTE. This enhancement suggests that ozonation may have chemically modified terpenoid constituents, possibly improving their interaction with the lipase active site and thereby increasing their inhibitory potency. Many studies and clinical cases have evidence to support that tea polyphenols show good antioxidant, anti-inflammatory, anti-cardiovascular disease, potential anti-obesity, anti-diabetic and other properties [40,41]. Moreover, ozonation of natural oils enhanced their potential anti-obesity effect inferred from pancreatic lipase inhibition [17].

### 2.8. Antioxidant Potential of Green Tea Extract

**Figure 8:**
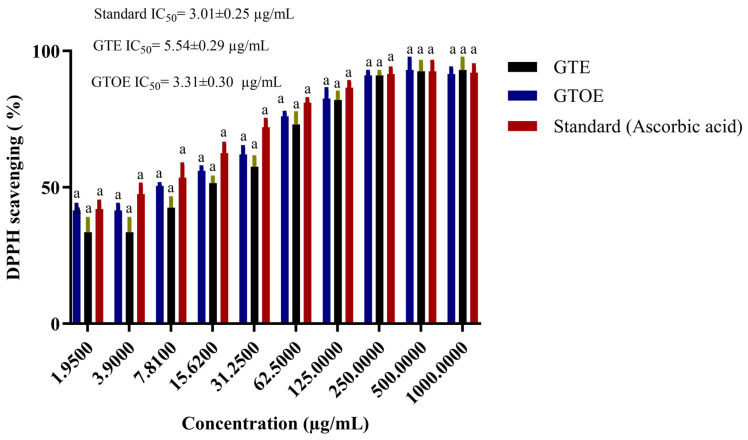
Statistical testing for DPPH percentages for GTE, GTOE versus standard. Data are presented as mean ± SD. Columns with similar letters indicate non-significant differences (*p* ≥ 0.05).

A parallel pattern was observed in antioxidant evaluation via DPPH scavenging. GTOE again outperformed GTE at all concentrations, with a pronounced increase evident even at moderate doses. By 62.5 µg/mL, GTOE exhibited over 77% scavenging activity, eventually reaching ~90% at 1000 µg/mL. Comparatively, GTE peaked at around 85%, while ascorbic acid reached 93%. The lower IC_50_ of GTOE (3.31 µg/mL) relative to GTE (5.54 µg/mL) confirms its improved radical scavenging efficiency (Figure 8). This enhancement may result from the formation of new oxidative metabolites during ozonation, known to exhibit heightened reactivity toward free radicals. Taken together, these findings indicate that ozonation not only augments the anti-obesity potential of green tea extracts through enhanced lipase inhibition but also elevates their antioxidant efficacy. The dual-functional behavior of GTOE, approaching that of synthetic standards, supports its prospective utility in formulations targeting obesity and oxidative stress-related complications. This synergistic enhancement underlines ozonation as a promising, non-toxic modification strategy to potentiate the bioactivity of natural extracts. Tea extract has excellent antioxidant activity because it is rich in polyphenols. Among these compounds, catechin compounds have strong antioxidant activity due to the large number of hydroxyl groups in their chemical structure [42,43]. The role of ozone to enhance the biological activities of plant extracts and its oil was reported previously [19]. Ozonation significantly enhanced the antioxidant capacity of peanut oil, as indicated by a reduction in IC_50_ values from 23.37 µg/mL in crude oil to 13.06 µg/mL post-ozonation [19]. Rafique et al. reported that 50% of methanolic green tea extract caused 80.82% of DPPH-radical scavenging activity [35]. Nevertheless, the extraction solvent and method can greatly influence antioxidant activity, as the solubility of phenolic and flavonoid compounds varies among ethanol, methanol, and water. Vareltzis et al. (2023) demonstrated that extraction conditions significantly affect antioxidant yield [44]. Therefore, ozone-assisted ethanol extraction represents a promising alternative that warrants further comparison with other medicinal plants, such as oregano and lavender.

## 3. Materials and Methods

### 3.1. Chemicals and Green Tea

The study’s materials, reagents were sourced from the Sigma-Aldrich (St. Louis, MO, USA). The consumables were purchased from BM-Egypt (Dokki, Giza, Egypt). Green Tea was obtained from Hala, CO (Cairo, Egypt). as an authenticated distributor in Egypt (202651). Six grams of green tea powder was mixed with 20 mL of ethanol in a 50 mL centrifuge tube, corresponding to a liquid-to-solid ratio of approximately 3.3 mL/g. This ratio was selected based on prior optimization experiments (unpublished data), which demonstrated that it provides an efficient balance between extraction yield and solvent use, ensuring maximal recovery of phenolic constituents without unnecessary dilution. The extraction was carried out for 24 h at room temperature (28 °C) with continues shaking. To minimize solvent loss, the tube was tightly sealed with parafilm and wrapped in aluminum foil to prevent evaporation and maintain stable extraction conditions.

### 3.2. Ozonation of Green Tea Extract

Ozone was produced using a corona discharge generator. The ozonation process was conducted by passing ozone gas through a Drechsel bottle containing 6 gm of green tea extract. The ozonation setup consisted of an ozone generator connected to a sealed glass reactor containing 100 mL of green tea extract. The reactor was placed in a cooling bath maintained at −8 °C to minimize thermal degradation of bioactive compounds during exposure. Ozone gas, generated from pure oxygen, was introduced at a controlled flow rate ranging from 0 to 7 L/min for a total duration of 5 h. The ozone concentration within the reaction chamber was continuously monitored, maintaining an average range of approximately 0.1-0.5 ppm. As the treatment progressed, the extract gradually thickened, indicating partial solidification. Upon completion, the ozonated extract was carefully collected, weighed, and stored in sealed containers at 5 °C for further analysis [20].

### 3.3. High-Performance Liquid Chromatography (HPLC) Analysis

Quantitative analysis of polyphenolic compounds in green tea extract was performed using an Agilent (Santa Clara, CA, USA) 1260 Infinity HPLC system equipped with a multi-wavelength detector. Chromatographic separation was achieved using a Zorbax Eclipse Plus C8 column (4.6 mm × 250 mm, 5 µm particle size). The mobile phase consisted of solvent A (water) and solvent B (0.05% trifluoroacetic acid in acetonitrile), delivered at a flow rate of 0.9 mL/min. The gradient elution program was set as follows: 0–1 min, 82% A; 1-11 min, 75% A; 11–18 min, 60% A; and then re-equilibrated back to 82% A from 18–24 min. The detection was carried out at 280 nm, with the column maintained at a constant temperature of 40 °C [45]. An injection volume of 5 µL was used for all samples. Data acquisition was managed using the Agilent ChemStation software platform (version LTS 01.11).

### 3.4. Evaluation of Antimicrobial Activity

The antimicrobial potential of GTE and GTOE were assessed against selected bacterial and fungal strains (*Aspergillus niger*, *Candida albicans*, *Bacillus subtilis*, *Staphylococcus aureus*, *Klebsiella pneumoniae*, *Salmonella typhi*) using the agar well diffusion method, along with determination of minimum inhibitory concentration (MIC) and minimum bactericidal/fungicidal concentration (MBC/MFC). Zone of inhibition was measured by introducing 100 µL of each sample into wells cut into Mueller-Hinton agar (for bacteria) or Sabouraud dextrose agar (for fungi), followed by incubation at 37 °C for 24 h for bacteria and at 30 °C for 72 h for fungi. Clear zones around the wells were recorded in millimeters. MIC values were determined using a broth microdilution method in 96-well plates, where serial dilutions of each extract were tested against microbial suspensions. MBC and MFC were identified by subculturing wells that showed no visible growth onto fresh agar plates and recording the lowest concentration that resulted in complete killing. Standard antibiotics [gentamicin (0.09 mg/mL)] and antifungal [fluconazole (0.28 µg/mL)] were used as positive controls [46].

### 3.5. Assessment of Antibiofilm Activity

The antibiofilm potential of GTE and GTOE were evaluated against *B. subtilis*, *S. aureus*, *K. pneumoniae*, and *S. typhi*, using a microtiter plate crystal violet assay. Bacterial cultures were grown overnight and adjusted to a standardized optical density (O.D_600_). In sterile 96-well plates, 100 µL of bacterial suspension was mixed with 100 µL of GTE or GTOE at different concentrations corresponding to 25%, 50%, and 75% of the minimum bactericidal concentration (MBC). Plates were incubated at 37 °C for 24 h to allow biofilm formation. After incubation, wells were gently washed with phosphate-buffered saline (PBS) to remove planktonic cells. The remaining biofilms were fixed with methanol, stained with 0.1% crystal violet, and then solubilized using 95% ethanol. Absorbance was measured at 570 nm to quantify biofilm biomass [21]. Percentage inhibition of biofilm formation was calculated in comparison to untreated control wells.

### 3.6. Time-Kill Kinetics Assay

The time-kill kinetics of GTE and GTOE were assessed against selected bacterial strains [*B. subtilis* (BS), *S. aureus* (SA), *K. pneumoniae* (KP), and *S. typhi* (ST)] using a standard broth microdilution method. Bacterial suspensions were prepared in Mueller-Hinton broth and adjusted to a final concentration of approximately 1 × 10^6^ CFU/mL. Each test tube contained bacterial culture along with GTE or GTOE at the minimum bactericidal concentration (MBC). Tubes were incubated at 37 °C with continuous shaking (150 rpm). At predetermined time intervals (0, 30, 60, 90, 120, 150, and 180 min), 100 µL aliquots were aseptically removed and serially diluted in sterile phosphate-buffered saline (PBS). Dilutions were plated on Mueller-Hinton agar and incubated at 37 °C for 24 h. Colony-forming units (CFUs) were counted to determine the number of viable bacteria at each time-point. The results were plotted as log_10_ CFU/mL versus time to construct time-kill curves [47]. A ≥3 log_10_ (99.9%) reduction in CFU/mL from the initial inoculum was considered indicative of bactericidal activity.

### 3.7. Evaluation of Anti-Hemolytic Activity

The protective effects of GTE and GTOE against bacterial-induced hemolysis were assessed using an erythrocyte lysis assay. A total of 10 mL of fresh human blood was collected from a healthy adult volunteer in normal condition, using EDTA tubes. The sample was centrifuged at 1500 rpm for 10 min to isolate red blood cells (RBCs). The procedure was performed in accordance with institutional ethical standards and was approved by the Ethics Committee of the University of Ha’il (Reference: H-2023-380). The RBC pellet was washed three times with sterile phosphate-buffered saline (PBS, pH 7.4) and then diluted to a 2% suspension. In sterile microtubes, 100 µL of bacterial culture (*B. subtilis*, *S. aureus*, *S. typhi*, and *K. pneumoniae*) was mixed with 100 µL of either GTE or GTOE at concentrations corresponding to 25%, 50%, and 75% of their minimum inhibitory concentrations (MICs). Then, 200 µL of the 2% RBC suspension was added to each tube. The mixtures were incubated at 37 °C for 1 h. As a positive control for complete hemolysis, RBCs were treated with 1% SDS. After incubation, the tubes were centrifuged at 1500 rpm for 10 min, and the absorbance of the supernatant was measured at 540 nm to quantify the released hemoglobin [19]. The percentage of hemolysis inhibition was calculated relative to the SDS-treated control.

### 3.8. Scratch Assay for Wound Healing Evaluation

The wound healing activity of GTE and GTOE was assessed using a scratch assay performed on human fibroblast HFB4 cells. Cells were seeded into 6-well plates and cultured in Dulbecco’s Modified Eagle Medium (DMEM) supplemented with 10% fetal bovine serum (FBS) until a confluent monolayer was formed. Once confluence was achieved, a uniform scratch (simulated wound) was made across the center of each well using a sterile 200 µL pipette tip. To remove detached cells and debris, wells were gently rinsed twice with sterile phosphate-buffered saline (PBS). Subsequently, cells were treated with serum-free medium containing either GTE, GTOE, or left untreated (control). Plates were incubated at 37 °C in a humidified incubator with 5% CO_2_ [48]. Images of the scratched area were captured at 0 h (immediately after scratching) and again after 48 h using a phase-contrast inverted microscope. The width and area of the scratch were measured using ImageJ software (version 1.54r). Wound closure was calculated as the percentage decrease in the wound area over time.

### 3.9. Assessment of Potential Anti-Obesity Activity via Pancreatic Lipase Inhibition

The inhibitory effect of GTE and GTOE on pancreatic lipase was investigated as an indicator of potential anti-obesity effect inferred from pancreatic lipase inhibition. The assay was carried out using porcine pancreatic lipase and *p*-nitrophenyl palmitate (*p*-NPP) as the substrate. A reaction mixture containing 0.1 mL of sample solution (GTE, GTOE, or standard inhibitor), 0.2 mL of enzyme solution (1 mg/mL in Tris-HCl buffer, pH 7.4), and 0.7 mL of Tris-HCl buffer was pre-incubated at 37 °C for 15 min. After pre-incubation, 1 mL of *p*-NPP substrate solution (dissolved in isopropanol and emulsified with gum Arabic) was added to initiate the reaction. The mixture was incubated for an additional 30 min at 37 °C. The reaction was terminated by placing the tubes in ice, and the absorbance was measured at 405 nm using a UV–Vis spectrophotometer [48]. Orlistat was used as a positive control. The percentage inhibition of lipase activity was calculated using the following equation:

Lipase inhibition (%) = [(A_*control* − A_*sample*)/A_*control*] × 100

where *A_control* is the absorbance of the control reaction (without sample), and *A_sample* is the absorbance in the presence of extract or standard.

All tests were performed in triplicate, and the IC_50_ values (concentration required to inhibit 50% of enzyme activity) were determined through linear regression analysis.

### 3.10. Determination of Antioxidant Activity Using the DPPH Radical Scavenging Assay

The free radical scavenging activity of GTE and GTOE was evaluated using the 2,2-diphenyl-1-picrylhydrazyl (DPPH) assay. A freshly prepared DPPH solution (0.1 mM) was made in methanol and kept in the dark for 30 min prior to use to ensure stability. Different concentrations of the test samples (GTE, GTOE) and the standard antioxidant (ascorbic acid) were prepared in methanol. For each reaction, 1.0 mL of the sample solution was mixed with 1.0 mL of the DPPH solution in a test tube. The mixture was vortexed briefly and then incubated in the dark at room temperature for 30 min. After incubation, the reduction in DPPH absorbance was measured at 517 nm using a UV–visible spectrophotometer. A blank containing methanol instead of the sample was used for calibration [49]. The ability of the sample to neutralize DPPH radicals was expressed as a percentage inhibition, calculated using the formula:

Scavenging activity (%) = [(A_*control* − A_*sample*)/A_*control*] × 100

where *A_control* is the absorbance of the DPPH solution without any antioxidant, and *A_sample* is the absorbance with the tested extract.

IC_50_ value (the concentration required to inhibit 50% of DPPH radicals) was estimated by plotting the percentage inhibition against concentration and analyzing the dose–response curve.

### 3.11. Statistical Examination

Each test was performed three times, and the average ± SD is shown. The *t*-test and one-way ANNOVA were utilized to distinguish among means utilizing the Graph Pad Prism V8 (CA, USA) software. Significant alterations were defined as results with *p* < 0.05.

## 4. Conclusions

The ozonation of green tea extract significantly enhanced its phytochemical profile, leading to superior performance across multiple biological assays. GTOE demonstrated stronger antimicrobial, antibiofilm, and anti-hemolytic activities, along with improved wound healing, antioxidant, and lipase inhibitory effects compared to the native extract. These results highlight ozonation as a powerful, natural strategy to boost the therapeutic efficacy of plant-based formulations, positioning GTOE as a promising candidate for use in food for postharvest treatment of fresh foods, surface decontamination and preservation. Besides, GTOE could possibly be used for tissue-repairing benefits in medicine and hygiene in medicine.

## Figures and Tables

**Table 1:** Detected compounds in green tea extract (GTE) and ozonized green tea extract (GTOE).

Detected Compound	GTE			GTOE		
	Area (mAU * s)	Retention Time	Concentration * (µg/g)	Area (mAU * s)	Retention Time	Concentration * (µg/g)
Gallic acid	871.62	3.57	3150.92 ^a^	889.50	3.57	3229.69 ^b^
Chlorogenic acid	156.63	4.28	1097.40 ^a^	155.53	4.28	1089.72 ^a^
Catechin	226.91	4.49	2634.09 ^a^	218.38	4.49	2535.09 ^a^
Caffeic acid	0.00	5.98	0.00 ^a^	48.48	6.06	145.11 ^b^
Syringic acid	257.72	6.45	820.94 ^a^	273.02	6.44	877.93 ^a^
Ellagic acid	435.65	7.36	2470.66 ^a^	489.62	7.35	2789.48 ^a^
Coumaric acid	0.88	9.15	1.61 ^a^	0.00	8.82	0.00 ^b^
Vanillin	15.27	9.38	20.85 ^a^	21.03	9.38	40.59 ^b^
Ferulic acid	41.59	10.06	120.25 ^a^	48.24	10.06	139.50 ^a^
Naringenin	1.67	10.57	8.07 ^a^	1.72	10.57	8.31 ^a^
Rosmarinic acid	8.76	12.32	35.90 ^a^	10.47	12.32	49.50 ^a^
Daidzein	11.77	16.32	33.64 ^a^	11.44	16.30	32.71 ^a^
Quercetin	17.86	17.50	112.65 ^a^	18.53	17.49	122.96 ^a^
Cinnamic acid	0.00	19.77	0.00 ^a^	9.42	19.87	10.20 ^b^
Kaempferol	31.60	20.91	161.98 ^a^	40.60	20.91	208.15 ^a^
Hesperetin	871.62	21.70	33.83 ^a^	17.61	21.70	41.63 ^a^

* Different letters above numbers refer to dramatic difference where (*p* ≤ 0.05).

**Table 2:** Antimicrobial properties of green tea extract (GTE) and ozonized green tea extract (GTOE) with estimated MIC and MFC values *.

Investigated Microorganisms	Zone of Inhibition (mm)			MIC (μg/mL)		MBC or MFC (μg/mL)	
	GTE	GTOE	Control	GTE	GTOE	GTE	GTOE
*B. subtilis*	13 ± 0.1	16 ± 0.3	20 ± 0.6	31.25 ±0.4	15.62 ± 0.2	125 ± 0.6	31.25 ± 0.1
*S. aureus*	19 ± 0.4	21 ± 0.7	15 ± 0.3	62.5 ± 0.6	15.62 ± 0.6	125 ± 0.6	31.25 ± 0.5
*K. pneumoniae*	16 ± 0.6	20 ± 0.5	15 ± 0.5	62.5 ± 0.3	15.62 ± 0.3	125 ± 0.6	15.62 ± 0.4
*S. typhi*	15 ± 0.8	22 ± 0.2	16 ± 0.3	31.25 ± 0.2	15.62 ± 0.2	62.5 ± 0.6	31.25 ± 0.3
*C. albicans*	13 ± 0.3	18 ± 0.1	21 ± 0.8	62.5 ± 0.6	31.25 ± 0.2	500 ± 0.6	125 ± 0.6
*A. niger*	NA	NA	30 ± 0.7	--	--	--	--

* Data are presented as means ± SD.

**Table 3:** Time-Kill Kinetics of GTE and GTOE Against Selected Bacterial Strain *.

Killing Kinetic Time (min)	BS		SA		KP		ST	
	GTE	GTOE	GTE	GTOE	GTE	GTOE	GTE	GTOE
0	38 × 10^5 a^ ± 1	38 × 10^5 a^ ± 1	24 × 10^5 a^ ± 1	24 × 10^5 a^ ± 1	19 × 10^5 a^ ± 2	19 × 10^5 a^ ± 2	25 × 10^5 a^ ± 1	25 × 10^5 a^ ± 1
30	150 × 10^3 a^ ± 3	17 × 10^3 b^ ± 2	172 × 10^3 a^ ± 1	121 × 10^3 a^ ± 1	125 × 10^4 a^ ± 1	110 × 10^4 a^ ± 1	211 × 10^4 a^ ± 2	174 × 10^4 a^ ± 2
60	113 × 10^2 a^ ± 2	21 × 10^2 b^ ± 1	27 × 10^3 a^ ± 2	142 × 10^2 b^ ± 1	171 × 10^3 a^ ± 2	197 × 10^2 a^ ± 1	19 × 10^3 a^ ± 2	32 × 10^2 b^ ± 1
120	154 × 10 ^a^ ± 1	14 ^b^ ± 1	30 × 10^2 a^ ± 2	230 ^b^ ± 1	124 × 10^2 a^ ± 2	180 ^b^ ± 1	16 × 10^2 a^ ± 3	194 ^b^
150	12 ^a^ ± 1	0 ^b^	160 ^a^ ± 1	47 ^b^ ± 1	25 × 10^2 a^ ± 2	36 ^b^	233 ^a^	0.0 ^b^
180	0.0	0.0	0.0	0.0	0.0	0.0	0.0	0.0

* Outcomes are represented as means ± SD where different letters (a) and (b) refer to significant difference *p* ≤ 0.05 among the two tested specimens. *B. subtilis* (BS), *S. aureus* (SA), *K. pneumoniae* (KP), and *S. typhi* (ST).

**Table 4:** Wound healing of GTE and GTOE *.

Treated HFB4	At 24 h		At 48 h		RM µm	Wound Closure % µm^2^	Area Difference
	Width	Area	Width	Area			
Control	1462.882 ^a^ ± 11	1,579,505.469 ^a^ ± 10	719.4707 ^a^ ± 16	776,768.5 ^a^ ± 13	15.48774 ^a^ ± 2	50.82205 ^a^ ± 1	802,737.0 ^a^ ± 14
GTE	1462.882 ^a^ ± 14	1,579,505.469 ^a^ ± 8	632.3957 ^a^ ± 14	682,776.5 ^a^ ± 9	17.3018 ^a^ ± 1	56.77276 ^a^ ± 2	896,728.9 ^a^ ± 13
GTOE	1462.88 ^a^ ± 12	1,579,505.469 ^a^ ± 7	568.6807 ^a^ ± 13	614,004.7 ^a^ ± 14	18.62919 ^a^ ± 4	61.12677 ^a^ ± 3	965,500.8 ^a^ ± 11

* Data are respected as means ± SD; similar letter above numbers refers to non-significant difference *p* ≥ 0.05 among treatments in the same column.

## Data Availability

Data presented in this study is contained within the article. Further inquiries can be directed to the corresponding author.
